# Investigation of the Self-Healing Behaviors of Microcapsules/Bitumen Composites by a Repetitive Direct Tension Test

**DOI:** 10.3390/ma9070600

**Published:** 2016-07-21

**Authors:** Jun-Feng Su, Peng Yang, Ying-Yuan Wang, Shan Han, Ning-Xu Han, Wei Li

**Affiliations:** 1 Department of Polymer Materials, School of Material Science and Engineering, Tianjin Polytechnic University, Tianjin 300387, China; YYW@163.com (Y.-Y.W.); shanhan@163.com (S.H.); weili2016@163.com (W.L.); 2 School of Navigational Engineering, Guangzhou Maritime Institute, Guangzhou 510725, China; pengyang@163.com; 3 Guangdong Provincial Key Laboratory of Durability for Marine Civil Engineering, Shenzhen University, Shenzhen 518060, China; nxhan@163.com

**Keywords:** bitumen, microcapsule, self-healing, tension test, strength

## Abstract

The aim of this work was to evaluate the self-healing behaviors of bitumen using microcapsules containing rejuvenator by a modified fracture healing–refracture method through a repetitive tension test. Microcapsules had mean size values of 10, 20 and 30 μm with a same core/shell ratio of 1/1. Various microcapsules/bitumen samples were fabricated with microcapsule contents of 1.0, 3.0 and 5.0 wt. %, respectively. Tension strength values of microcapsules/bitumen samples were measured by a reparative fracture-healing process under different temperatures. It was found that these samples had tensile strength values larger than the data of pure bitumen samples under the same conditions after the four tensile fracture-healing cycles. Fracture morphology investigation and mechanism analysis indicated that the self-healing process was a process consisting of microcapsules being broken, penetrated and diffused. Moreover, the crack healing of bitumen can be considered as a viscosity driven process. The self-healing ability partly repaired the damage of bitumen during service life by comparing the properties of virgin and rejuvenated bitumen.

## 1. Introduction

Bitumen can be defined as a self-healing material because it has the potential to restore stiffness and strength by closing microcracks that occur when pavement is subjected to traffic loads or under high temperature [1]. The crack repair mechanism in an asphalt pavement system is attributed to the wetting and inter-diffusion of material between two microcrack faces to achieve the original material properties [2]. However, this capability decreases or disappears because bitumen ageing leads to pavement failure after years of usage, including surface raveling and reflective cracking [3]. Asphalt concrete stiffness increases, whereas its relaxation capacity decreases. Then, the binder becomes more brittle, causing the development of microcracks, and ultimately the interface debonding between aggregates and the binder [4].

As in nature, the self-healing performance of asphalt pavements can be improved through the methods of particle addition, polymer blending, heat induction, and rejuvenator usage [5]. In all these methods, it has been found that rejuvenator usage is the only method that can restore the original properties of pavements [6]. Rejuvenating agents are able to reconstitute the binder’s chemical composition and consist of lubricating and extender oils that contain a high proportion of maltene constituents [7]. In recent years, the microencapsulated rejuvenator has been used to overcome the poor penetration ability of oily rejuvenator through the bitumen surface [8]. In previous work, microcapsules containing rejuvenator have been prepared by in suit polymerization using a methanol-modified melamine-formaldehyde resin as shell material [8,9,10]. The microcapsules have a satisfactory thermal stability in bitumen and reliable mechanical properties. During bitumen aging, punctured microcapsules could leak oily liquid rejuvenator into microcracks. Capillary motion fills the cracks with rejuvenator at a speed that is determined mainly by the bitumen microcapsule volume [11]. Such a diffusion phenomenon has been observed using a fluorescence microscope. This product may be an environmentally friendly powder-encapsulating suitable-size rejuvenator for chemical and construction engineering applications [12].

**Figure 1:**
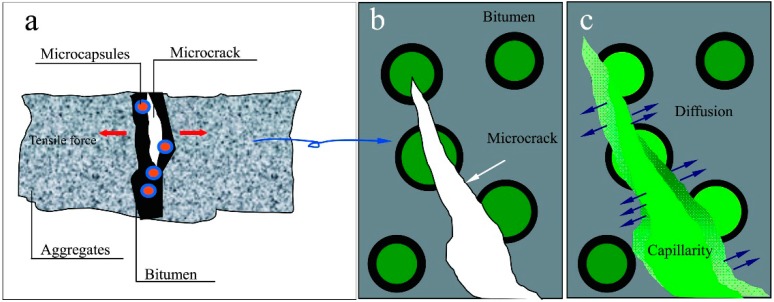
Illustration of the self-healing process of aged bitumen by microcapsules containing rejuvenator: (**a**) the structure of asphalt consisting of bitumen and aggregates; (**b**) microcrack generation and microcapsules broken; and (**c**) the self-healing of aged bitumen by leaked rejuvenator with the help of diffusion and capillarity actions.

The main issue in self-healing bitumen is to develop asphalt pavement material that can heal itself without external intervention. Our goal is to develop an asphalt pavement material that can mimic nature repeatedly. We would therefore like to design asphalt pavements with multi-self-healing action. Without this multi-self-healing ability, pavements are vulnerable in areas without rejuvenator, because cracking after initial healing may continue without healing the damage. This leads to the reoccurrence of asphalt pavement failure. In a previous study, it was reported [12] that multi-self-healing behavior occurs when wetting and diffusion are achieved repeatedly. Figure 1 illustrates the self-healing of aged bitumen by microcapsules that contain rejuvenator, with the help of diffusive and capillary actions. Asphalt concrete weakening occurs primarily because of a deterioration of bituminous binder film, which acts as glue, and may fail by loss of cohesion within the binder and/or loss of adhesion between the binder and aggregates [13]. When rejuvenator fills the microcrack by capillary action, diffusion causes the aged bitumen to soften, and there is a small probability for crack reoccurrence. Based on this mechanism of self-healing, it can be deduced that the microcrack trigger depends on the degree of bitumen softness and microcapsule breakage depends on the point stress value of the microcrack [11].

Tensile fracturing is one of the main failure behaviors of bitumen as films between mineral aggregates. Therefore, the effect of tensile stress on bitumen is essential to understand its self-healing properties. We may be able to find a suitable tensile fracture method to prove that microcapsules provide the recovery action for multi-self-healing of aged bitumen. The healing phenomenon of bituminous materials occurs by visco-elastic and viscous healing [5,14]. Moreover, bitumen properties are highly temperature- and time-dependent. Hence, bitumen self-healing is a complex process, which is dependent on the rest time between two load pulses, the temperature, crack phase, and material type. Normally, self-healing is evaluated by fracture tests at various times [15]. Several mechanical methods have been reported to measure bitumen self-healing, including the discontinuous fatigue test with various rest times/load periods, the fatigue-healing-refatigue test, the intrinsic two-piece healing test, and the fracture-involved healing test [5]. Research has focused mainly on self-healing behavior during repetitive loads. For example, Hammoum [16] established a repeated fracture test method to investigate the self-healing of pure bitumen using two hemispheric protuberances, which can simulate aggregates in the asphalt mixture. A controlled tensile loading was applied with a displacement speed of 12.5 μm·min^−1^. After healing, another load was applied. The bitumen could almost recover its original properties from an analysis of the loading-reloading curves. Poulikakos [13] investigated brittle and ductile regimes through the tensile behavior of two types of visco-elastic bituminous films confined between mineral aggregates or steel as adherents. Uniaxial specimens were fabricated using a prototype setup that allows for the construction of micro-scale thin films and a visualization of failure phenomena. To reduce limitations of time-consuming and operational complexity, Qiu [2] reported a simple self-healing test procedure, in which a fast displacement speed loading was applied first to produce a flat open crack with 100–200 μm width. Healing, in this case, is believed to be viscosity driven, and consists of two steps, namely crack closure and strength gain. This testing procedure was simple and effective to evaluate and compare self-healing of bituminous materials. However, these methods were not all focused on investigating the repetitive capability of bitumen. In addition, no knowledge can be applied to establish the feasibility of microcapsules that contain rejuvenator to recover aged bitumen.

The aim of this paper is to evaluate the novel mulit-self-healing behaviors of bitumen using microcapsules containing rejuvenator by a modified fracture-healing-refracture method using a repetitive tension tests. Pure bitumen binder does not exist in a real pavement, rather a combination of bitumen, filler and additives known as mastic. No mineral fillers ere mixed in bitumen in order to simply and unravel the tensile fracture and healing behaviors in this study. The tension strength values were compared to calculate the recovery efficiency of self-healing behaviors. Morphologies were observed to help understand the healing mechanism and supply more information about microstructure. A simple mechanism was given based on the healing data considing the factors of time, temperature, and content of microcapsules, which will guide the controlment of microcapsule structure and the useage condations of microcapsules in bitumen.

## 2. Experimental

### 2.1. Materials

The shell material of microcapsules was commercial prepolymer of melamine-formaldehyde modified by methanol (MMF) (solid content was 78.0%) purchased from Aonisite Chemical Trade Co., Ltd. (Tianjin, China). Styrene maleic anhydride (SMA) copolymer (Scripset^®^ 520, Hercules, CA, USA) was applied as dispersant. The core material used as rejuvenator is dense, aromatic oil (density, 0.922 g/cm^3^, viscosity, 4.33 Pa·s, under 20 °C) obtained from Petro plus Refining Antwerp (800DLA, Antwerp, Belgium). The bitumen used in this study was 80/100 penetration grade obtained from Qilu Petrochemical Industries Co. (Jinan, China). The 40/50 penetration grade bitumen was considered as aged bitumen in this study, which was artificially produced by a thin film oven test [5,9].

### 2.2. Microcapsule Fabrication Process

The method of fabrication microcapsules containing rejuvenator by coacervation process has been reported in our previous work [8]. It can be divided into three steps:

1. SMA was added to 100 mL water at 50 °C and allowed to mix for 2 h. Then, a solution of NaOH (10%) was added dropwise, adjusting its pH value to 10. The above surfactant solution and rejuvenator were emulsified mechanically under a vigorous stirring rate for 10 min using a high-speed disperse machine.
2. The encapsulation was carried out in a 500 mL three-neck round-bottomed flask equipped with a condensator and a tetrafluoroethylene mechanical stirrer. The above emulsion was transferred in the bottle, which was dipped in a steady temperature flume (room temperature). MMF prepolymer was added dropwise with a stirring speed of 500 r·min^−1^. The temperature was increased to 80 °C with a rate of 2 °C·min^−1^.
3. After polymerization for 1 h, the temperature was decreased slowly to ambient temperature. At last, the resultant microcapsules were filtered and washed with pure water and dried in a vacuum oven.

### 2.3. Mixing of Bitumen and Microcapsules

The 40/50 aged bitumen was blended with different microcapsules using a propeller mixer for 30 min at 160 °C with a constant speed of 200 r·min^−1^. 

### 2.4. Morphology Observation

The dried microcapsules were observed by using an Environmental Scan Electron Microscopy (ESEM, Philips XL30, Czech Republic) at an accelerated voltage of 20 kV. Self-healing behaviors of bitumen were observed by a fluorescence microscope (CKX41-F32FL, Olympus, Japan). As bitumen is a temperature-sensitive material, the observation is in an environment of 0 °C temperature.

### 2.5. Mean Size of Microcapsules

The mean size of microcapsules was measured by a laser particle size analyzer (HELOS-GRADIS, SYMPATEC GMBH, Germany).

### 2.6. Direct Tension Test

**Figure 2:**
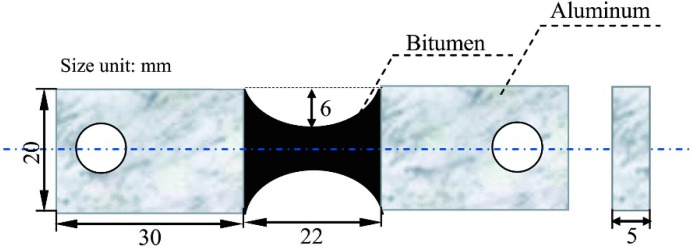
Illustration of tension test sample size.

**Figure 3:**
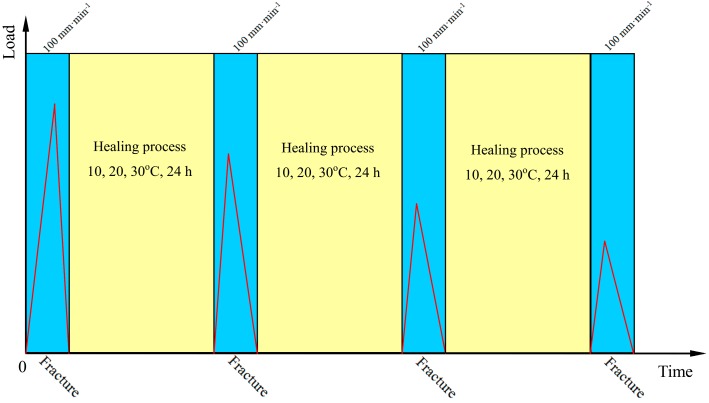
Illustration of the relationship between the time and fracture with a repetitive tension test.

- Sample preparation: the testing samples were prepared in a pre-heated silicone rubber mould. Figure 2 shows the size of the tension test sample. The geometry of the bitumen can ensure the stress concentration in the middle of the bitumen sample [2]. Each sample was covered with another flat pre-heated silicone rubber in order to ensure the bitumen has the same shape on both sides. After being de-moulded, the samples were conditioned in a chamber for 24 h at 0 °C.
- Fracture and healing: samples were tested by a high–low temperature tension test machine (FR-103G, Farui Tech Co. Ltd., Shanghai, China; ±2.5% RH, ±1 °C) using a displacement speed of 100 mm·min^−1^ at 0 °C. The middle was fractured under the direct tension. The two parts of one broken sample were immediately put into the module again to ensure the two parts fit the crack very well. Each sample in the module was heated for 24 h under various temperatures of 10, 20 and 30 °C.
- Re-healing: After a healing process, the sample was re-conditioned in a chamber for 24 h at 0 °C. Then, the sample was de-moulded and the fracture process was repeated with the displacement speed of 100 mm·min^−1^ at 0 °C. The above re-fracture and re-healing rounds were repetitively tested, and the data were recorded automatically. Figure 3 illustrates the relationship between the time and fracture loads.

### 2.7. Characterization of Virgin and Rejuvenated Bitumen

The properties of virgin bitumen, aged bitumen and rejuvenated bitumen were tested including penetration (ASTM D5 [17]), softening point (ASTM D36 [18]) and viscosity (ASTM D4402 [19]). Five samples with the same state were mixed homogenously to test the properties. For example, to test the penetration property of aged bitumen after two tensile test cycles, five samples (after two tensile test cycles) were heated and mixed to form one big sample. Then, the penetration was tested under 25 °C.

### 2.8. Evaluation of the Self-Healing Capability

The self-healing capability of bitumen was calculated by the penetration value divided by the last-time penetration value for the same sample,

(1) $$SH=\frac{\sigma_{i}}{\sigma_{i+1}}\times 100\%$$

where *SH* is the self-healing percentage demonstrating the self-healing capability, *σ_i_*_+1_ is the penetration (softening point and viscosity) value, *σ_i_* is the last-time penetration (softening point and viscosity) value, and *i* is the fracture times as 1, 2, 3 and 4 in this study.

## 3. Results and Discussion

### 3.1. Microcapsule/Bitumen Sample Characterization

**Figure 4:**
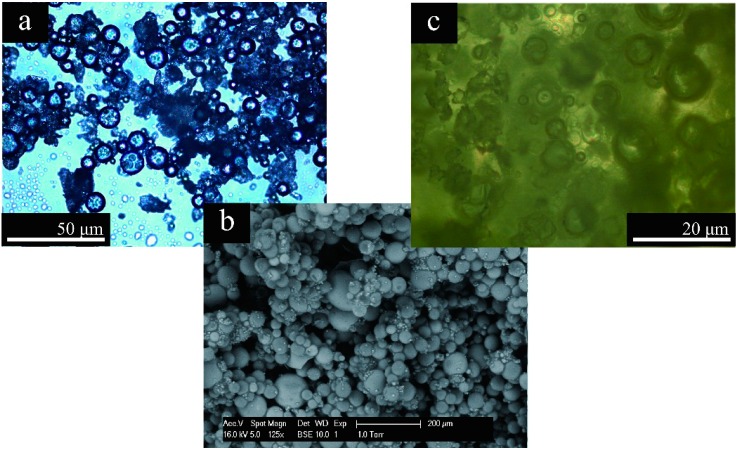
Morphologies of microcapsules/bitumen samples; (**a**) optical microscope morphology of microcapsules in emulsion; (**b**) SEM morphology of microcapsules and (**c**) microcapsules/bitumen composite sample.

MMF resin can be successfully applied to fabricate microcapsules containing rejuvenator by the in situ polymerization method [8]. Moreover, the shell thickness, surface morphology and average size of microcapsules can be controlled by regulating the core/shell ratio, prepolymer adding speed and emulsion stirring rate [10]. The microcapsules were fabricated with a core/shell ratio of 1/3 fabricated by 3000 r∙min^−1^ emulsion stirring rates. Figure 4a shows the optical microphotographs of microcapsules illuminating the encapsulation details. In this study, SMA molecules were used as a disperser hydrolyzing in water by NaOH and forming carboxyl (–COOH) groups. These hydrophilic polar groups associated with water molecules and trimly covered the oil droplets’ surface; then, hydrophobic groups oriented into oil droplets and hydrophilic groups out of oil droplets [8]. It is observed that the organic core material is dispersed into particles in water and finally formed microcapsules with a mean sizes are about 25 μm. Figure 4b shows the dried microcapsules with regular globe shape with smooth surfaces. The microcapsules keep the regular global shape and the shells are compact without holes and cracks. There is no adhesion and impurity substance between microcapsules. Figure 4c shows the original state fluorescence morphologies of microcapsules dispersing in bitumen (3.0 wt. %). The microcapsules had survived in the bitumen and the microcapsules homogeneously dispersed in bitumen. In addition, the microcapsules retained their global shape with no cracks or thermal decomposition. These results indicate that microcapsules can resist the thermal effects of asphalt during common application. 

**Figure 5:**
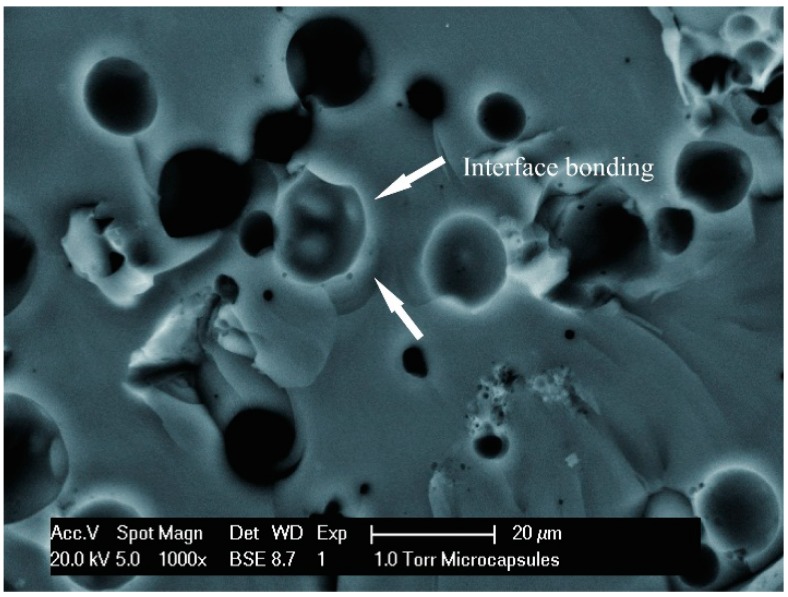
SEM cross-section morphology of a microcapsules/bitumen sample.

It has been reported in our previous work [9] that the decomposition temperature of these microcapsules is higher than 300 °C. This degradation temperature was higher than the melting temperature of bitumen (180 °C). It indicates that the cured MMF resin will not be thermally decomposed during mixing with melting asphalt. With the temperature increasing, the shell will firstly crack or break under high temperature before decomposition. It also been confirmed [12] that the mechanical properties of microcapsules are strong enough to remain intact during manufacturing and further processing, such as drying, pumping, and mixing. Another important issue to be considered is the bonding between microcapsules and bitumen. It is found that microcracks and interface separation may appear in a repeated vigorous thermal absorbing releasing process for microcapsule/matrix composites [20]. During a repeated temperature change process with heat transmission, expansion and shrinkage may occur in both of microcapsules and bitumen due to the different expansion coefficients. The tensile strength will be greatly affected by the debonding microstructure between filler and matrix. Figure 5 shows the SEM cross-section morphology of a microcapsules/bitumen sample. It is clear that the microcapsules have compact structure and global shape in bitumen. No interface debonding emerged between microcapsules and bitumen after a mixture and a temperature change. An interphase region is comprised of polymer molecules that are bound at the filled particles surface, and they exhibit unique physical and chemical properties [21]. 

Table 1 lists the structure parameters of microcapsules/bitumen samples including core/shell ratio, content and mean size of microcapsules. The mean size of microcapsules can be adjusted by controlling the stirring rate during emulsion step of core material. Higher stirring rates disperse the core material into smaller droplets [8]. To simplify the complex system, the core/shell ratio of microcapsules is 1/1 and the microcapsules contents are 1.0, 3.0 and 5.0 wt. %, respectively. Three types of microcapsules were selected with the mean size of 10, 20 and 30 μm. The mean size is an important parameter for microcapsules containing rejuvenator influencing their application possibility in asphalt. The reason is that too small of a size may limit the encapsulated content of rejuvenator. On the other hand, thin asphalt films between aggregates are less than 50 μm, and the size of microcapsules containing rejuvenators should be smaller than 50 μm to avoid being squeezed or pulverized during asphalt forming [10]. 

### 3.2. Tension Tests of the Self-Healing Microcapsule/Bitumen Samples

It has been found that when portions of fractured bitumen make contact again, the bitumen healing periods vary [13]. When two parts of bituminous binders make contact, the interface disappears due to the molecular mutual confluence and molecular chain entanglement. The results also indicate that rejuvenators with different ranges of viscosity influence the penetration properties of binder and properties of the structural performance of recycled asphalt mixtures. A conclusion on the properties of recycled mixtures using rejuvenator with different components indicates that aged binders can be recovered to a target penetration using different rejuvenators if an adequate amount is added [22]. The encapsulation ratio of rejuvenator in microcapsules in this study is ~80%–85%, which has been calculated in our previous work [8]. Thus far, it can be deduced that the number of broken microcapsules in interfaces will determine the healing ability of bitumen in this microcapsule/bitumen system. Because microcapsules in the interface may not break simultaneously during repetitive multi-healing, the rejuvenator will be supplied continuously. Therefore, the rejuvenator mass will be a main factor that influences this process, and it is determined by the mean size and microcapsule content in bitumen.

**Figure 6:**
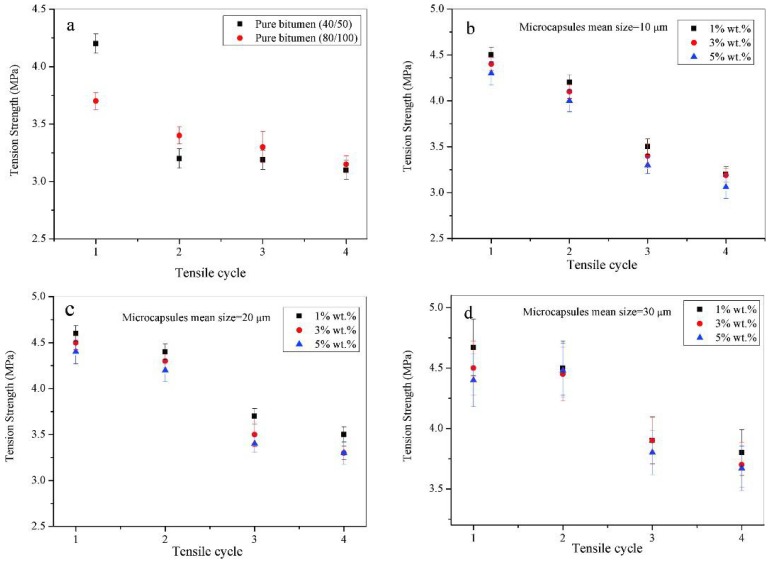
Tension strength values of microcapsules/bitumen samples during a reparative fracture-healing process (four recycles) under temperature of 0 °C, microcapsules contents are 1.0, 3.0 and 5.0 wt. %; (**a**) tension strength values of pure bitumen (80/100) and bitumen (40/50), tension strength values of bitumen samples with microcapsules mean size of; (**b**) 10 μm; (**c**) 20 μm; and (**d**) 30 μm.

Figure 6 shows the tensile strength values of microcapsule/bitumen samples during reparative fracture-healing (four recycles) at 0 °C, and for a microcapsule content of 1.0, 3.0, and 5.0 wt. %. The microcapsules had mean sizes of 10, 20, and 30 μm. Multiple studies have indicated the importance of proper rejuvenator dose and its effect on binder and mixture properties. However, the dose must be balanced to ensure a reduced stiffness and improved resistance to fracture without over-softening of the binder to cause rutting. In Figure 6a, pure 40/50 and 80/100 bitumen samples have tensile strengths of 4.2 and 3.7 MPa. Because of the increasing bitumen stiffness, the tensile strength has increased with a decrease in tensile elongation. After the healing cycles, both samples have almost the same tensile strength of ~3.1 MPa. This phenomenon may be attributed to bitumen aging in the interface according to a multi-fracture-healing process. Figure 6b shows the tensile strength values of bitumen (40/50) samples containing microcapsules (10 μm) with 1.0, 3.0, and 5.0 wt. %. The sample of 5.0 wt. % has a large tensile strength, which may be attributed to particle reinforcement [23]. The tensile strength of each sample decreases with increasing tensile cycles. After the first fracture-healing process, samples with microcapsule contents of 1.0, 3.0, and 5.0 wt. % have a tensile strength of 4.5, 4.4, and 4.3 MPa, respectively. All values are larger than pure bitumen (80/100) under the same condition. Even after four fracture-healing cycles, they still have tensile strengths of 3.2, 3.2, and 3.1 MPa. Figure 6c shows the tensile strengths of bitumen (40/50) samples with microcapsule (20 μm and 30 μm) contents of 1.0, 3.0, and 5.0 wt. %. Larger microcapsules with the same content can supply more rejuvenator into bitumen during fracturing. After the first tensile fracture-healing cycle, bitumen samples with 20 μm microcapsules have tensile strengths of nearly 4.5 MPa. After four tensile fracture-healing cycles, these samples have tensile strengths of ~3.5 MPa, which is larger than the data of pure bitumen under the same conditions. Rejuvenator, therefore, leaked out of microcapsules and enhanced aged bitumen tensile properties. The same trend also exists for bitumen that contains microcapsules with a mean size of 30 μm as shown in Figure 6d.

Traditionally, the number of loading repetitions required to achieve a 50% reduction in initial stiffness is considered to be an appropriate parameter to evaluate fatigue performance [2,5]. The data imply that the rejuvenated bitumen has a longer fatigue life compared to the aged bitumen. Normally, rejuvenator has a natural ability to diffuse rapidly into bituminous binder and mobilize aged asphalt. It can make the binder softer and produce a workable mixture that can be paved easily and compacted to the required density. Different rejuvenators do not have the same penetration ability in aged bitumen. Although it is not possible to compare directly, these results show somewhat that binders with higher asphaltene content have a lower stiffness at lower temperature.

### 3.3. Effect of Temperature and Time on Self-Healing Capability

Bitumen can be considered as a colloidal suspension of asphaltenes particles in an oily continuous maltenes (matrix) containing resin. In bitumen, oils have the lowest molecular weight, resins intermediate, and asphaltene particles have the highest molecular weight [11]. The viscoelastic behavior and mechanical properties of bitumen strongly depend on temperature attributing to the bituminous binder. Moreover, binders determined the cohesion, adhesion and durability of the bituminous materials. The viscoelastic nature also means that they soften when heated and harden under low temperature. Therefore, it can be imagined that the addition of oily rejuvenator in aged bitumen will greatly influence the mechanical properties under various temperatures. The rejuvenator action in bitumen can be understood by comparing the mechanical properties of pure bitumen and microcapsules/bitumen.

**Figure 7:**
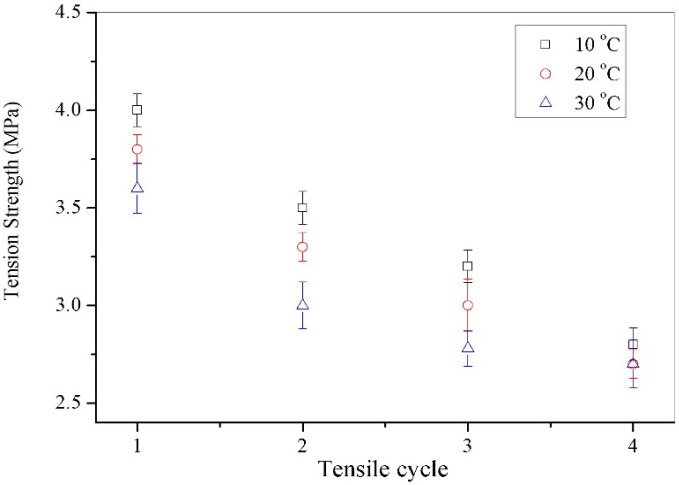
Tension strength values of microcapsules/bitumen samples (microcapsules: 3.0 wt. %, 20 μm) during a reparative fracture-healing process (four recycles, each healing time: 24 h) under different temperatures.

Figure 7 shows the tension strength values of microcapsules/bitumen samples (microcapsules: 3.0 wt. %, 20 μm) during a reparative fracture-healing process (four recycles, each healing time: 24 h) under different temperatures of 10, 20 and 30 °C, respectively. Firstly, the sample has less tensile strength because of the viscoelastic properties under higher temperature. After the first fracture-healing cycle, the samples have tensile strength values of 4.0, 3.8 and 3.6 MPa, respectively. After four fracture-healing cycles, the samples have tensile strength values of 2.8, 2.7 and 2.7 MPa. From a point of molecular structure, the supplement of little molecules into bitumen has decreased the resistance for long-chain molecules regulating their states. The rejuvenator penetration into bitumen has definitely reduced the impact of temperature after four cycles.

**Figure 8:**
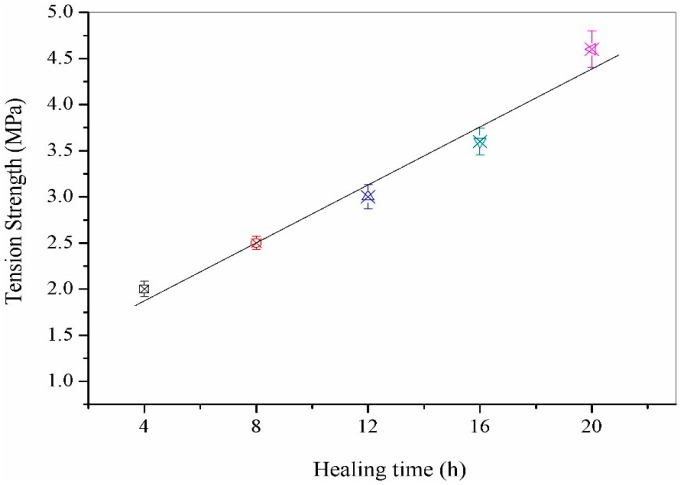
Tension strength values of microcapsules/bitumen samples (microcapsules sample-5, 3.0 wt. %, 20 μm) during a fracture-healing process under 10 °C with healing time of: 4 h, 8 h, 12 h, 16 h and 20 h.

The bitumen need to be considered as a viscoelastic material such as creeping under a constant loading or relaxing under an imposed straining. It indicates the stress-strain relationship in a direct tension test combining two major factors of the damage development and relaxation. Qiu [5] had investigated the time effect on the self-healing percentage of pure bitumen by a tensile test. It was considered that an initial self-healing mechanism can be related to the wetting, and then a further increase in time of the self-healing ratio was attributed to a diffusion process. Figure 8 shows the tension strength values of microcapsules/bitumen samples (microcapsules: 3.0 wt. %, 20 μm) during a fracture-healing process under 10 °C with a healing time of 4, 8, 12, 16 and 20 h. The tensile strength value has a linear trend with the increasing of time. The reason is less wetting in the short term and longer diffusion is needed as observed in this test. With the increase in healing time, the reloading energy has been enhanced. Moreover, a strength recovery obeys the rules of time-temperature superposition principle [5]. 

### 3.4. Properties of Virgin and Rejuvenated Bitumen

It has been reported that the majority of the encapsulated rejuvenator can leak out of the microcapsules and penetrate into the aged bitumen [11,12]. The subsequent rejuvenating effect can be assessed by comparing the properties of aged bitumen samples under the same conditions [15]. To this purpose, a series of microcapsules/bitumen samples were first heated at 200 °C for 12 h and then stored for 60 days. Efficiency of the rejuvenator depends on its viscosity and the quantity added to the aged bitumen, and thus we decided to conduct a preliminary investigation to explore the effects of blending known quantities of rejuvenator with penetration grade bitumen on binder rheology. 

In this study, the original bitumen (80/100) had penetration, softening point and viscosity values of 86 d-mm, 46.7 °C and 325 mPa·s. In contrast, the aged bitumen (40/50) had values of 43 d-mm, 53.5 °C and 578 mPa·s. The aged bitumen was subsequently rejuvenated with microcapsules at 3.0% by weight of bitumen (microcapsules: 20 μm). Table 2 lists the penetration, softening point and viscosity of the aged bitumen before and after rejuvenation with varying self-healing cycles. After the healing cycles, all of the properties of aged bitumen had been partly returned to its original state. Even with four-time healing cycles, the aged bitumen still can not be softened as the original state. In other words, the addition of approximately 3.0% microcapsules returns the aged 40/50 bitumen to a condition similar to that of the original bitumen. This result is attributed to a reduction in the ratio of asphaltenes to maltenes. In the aged bitumen, increases in the level of high molecular weight asphaltenes tend to produce a harder material with lower temperature susceptibility and thus increase the softening point. The addition of the rejuvenator, however, is evidently capable of mitigating this effect. The same workability is expected for the rejuvenated bitumen. Usually, material properties degrade over time because of the initiation of damage on a microscopic scale. This damage tends to grow and ultimately leads to a failure. A conclusion can be drawn that the self-healing capability of bitumen partly repairs the damage during service life. It must be mentioned that the self-healing ability connects with the interface energy. A lower energy can not activate a crack healing because less energy can not promote the molecule movement. Besides the temperature, time also has a great effect on the properties of rejuvenated bitumen.

**Figure 9:**
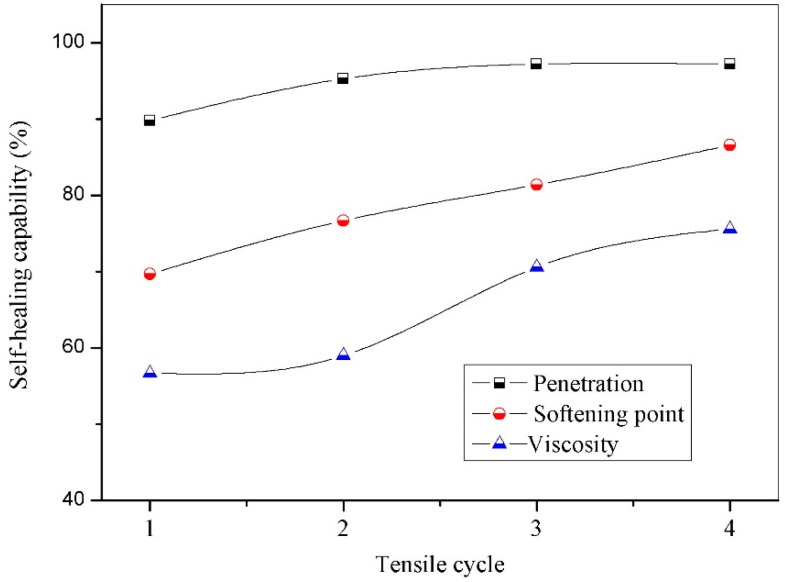
Self-healing capability of microcapsules/bitumen samples (microcapsules: 3.0 wt. %, 20 μm) calculated by properties values (penetration, softening point and viscosity) during a reparative fracture-healing process (four recycles) under 0 °C, each cycle healing time of 24 h.

Figure 9 shows the self-healing capability values of microcapsules/bitumen samples (microcapsules: 3.0 wt. %, 20 μm) calculated by penetration, softening point and viscosity data, respectively. During a four reparative fracture-healing process under 0 °C, each cycle had the same healing time of 24 h. The self-healing capability based on penetration had not changed too much after the four tensile cycles. The reason is that the diffusion behaviours of bitumen have great relationships with temperature, viscosity of the diffusion medium, diffusant size and polarity. In previous work [12], the diffusion direction of rejuvenator in aged bitumen can be identified. As the penetration data were tested arbitrarily on the surface of bitumen, some areas still did not soften.

However, the recovery percentage of properties based on softening point and viscosity both have an obvious increase. A model is called the Physicochemical theory, which can be used to explain the above data changes based on the molecular level. Based on the results of all of our research [12], microcapsules containing rejuvenator have been considered as a positive product to recovery the properties of the aged bitumen. In this process, the penetration and diffusion make a role of determining the microstructure of bitumen. The diffusing ability of rejuvenator in aged bitumen can be enhanced through increasing temperature or prolonging time. However, the diffusing behavior is restricted by the volatilization of light components and aging degree of bitumen. Another interesting issue in this microcapsule/bitumen system is that the microcapsules can be broken at various times during the aging process of bitumen because the microcapsules have different shell thickness and size [24]. It means that it will provide a continuous supply of rejuvenator into aged bitumen in a long service history. With a constant change and adjustment of microstructure, viscosity and thixotropy of bitumen, it possesses a healing ability numerous times.

### 3.5. Morphology Analysis of a Tension Fracture

**Figure 10:**
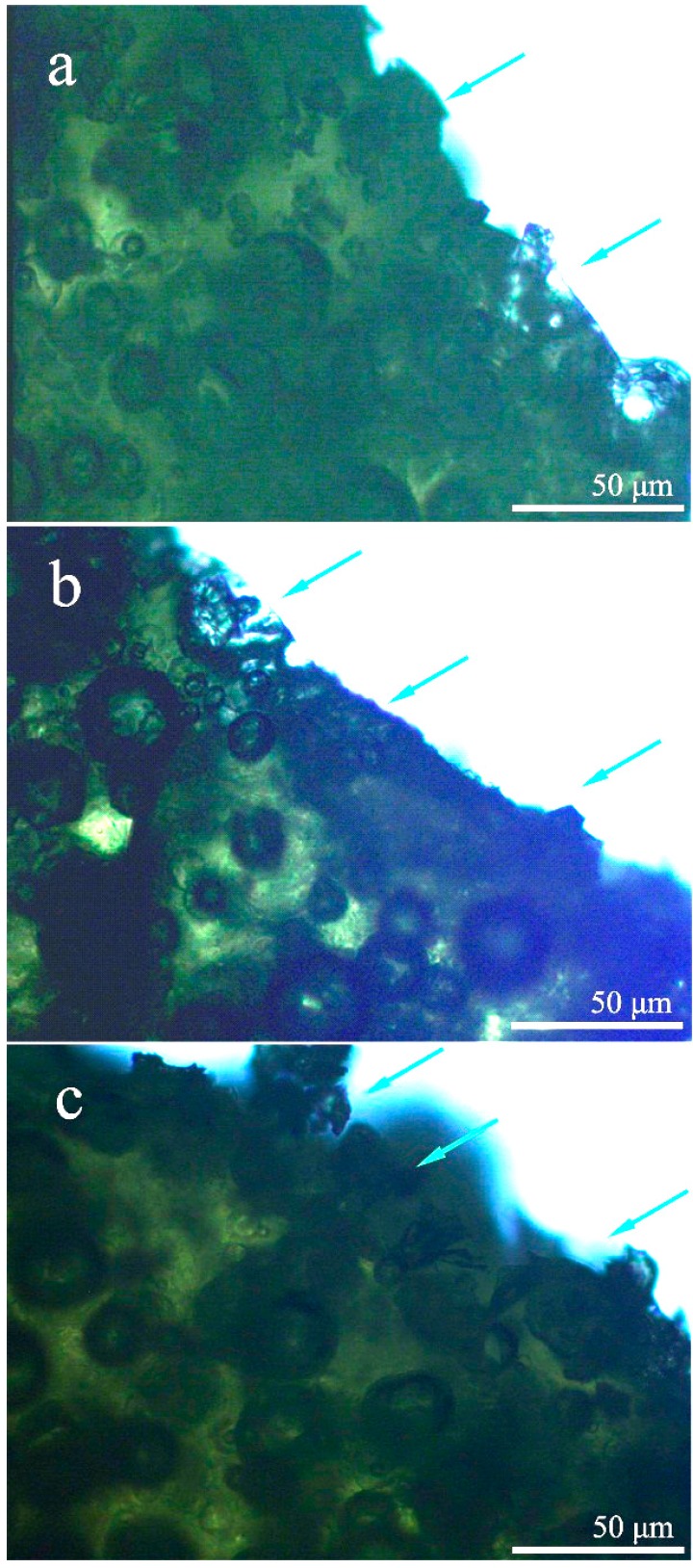
Morphologies of broken microcapsules on the surface of cracks under temperature of (**a**) 0 °C; (**b**) 10 °C; and (**c**) 20 °C.

Morphology changes are usually used to give some details of structure variation to analyze the self-healing capability of bitumen. On the other hand, morphology observation can help to confirm that the healing of microcapsules/bitumen composites is coming from the microcapsules, but not the natural ability of bitumen. Figure 10 shows the optical microscope morphologies of broken microcapsules by bitumen microcracks generated under temperature of 0, 10 and 20 °C, respectively. As the microcapsules have good bonding with bitumen, the crack can easily break the microcapsules. Under low temperature of 0 °C, as shown in Figure 10a, the shell has a brittle fracture with sharp edges and corners. When the temperature was increased from 0 to 30 °C as shown in Figure 10b,c, the microcapsules had an elastic tearing behavior by the microcrack. This phenomenon accords with the results of mechanical testing of single microcapsule owning elastic-plastic deformation and break behaviors.

**Figure 11:**
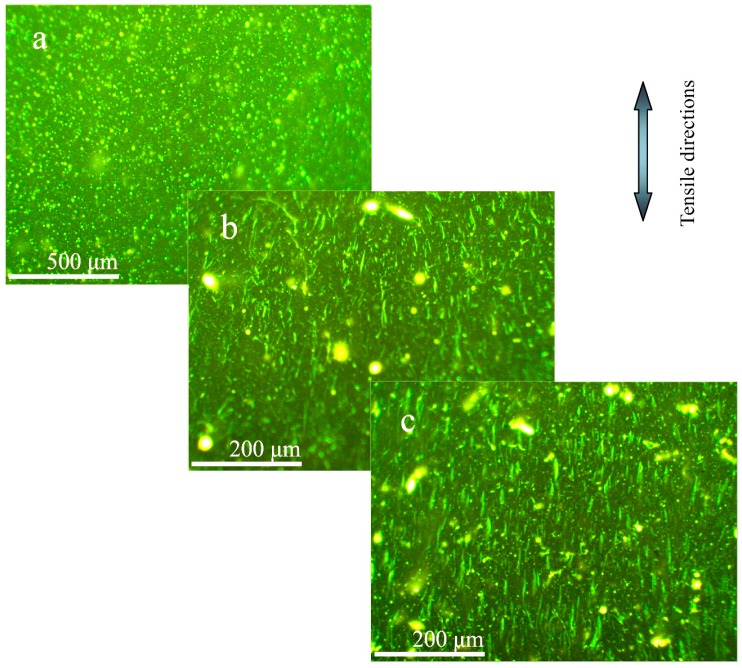
Fluorescence microscopy morphologies of microcapsules/bitumen sample (bitumen, 40/50; microcapsule, 20 μm, 3.0 wt. %) of (**a**) original; (**b**) after going through the healing cycle once and (**c**) going through the healing cycle twice.

Figure 11 shows the fluorescence microscopy morphologies of microcapsules/bitumen sample (bitumen, 40/50; microcapsule, 20 μm, 3.0 wt. %) of the original, after one healing cycle and two healing cycles, respectively. The arrow points to the tensile directions of microcapsules/bitumen sample. As shown in Figure 11a, it can be seen that the microcapsules dispersed in bitumen homogeneously. In Figure 11b, the tension process has broken the microcapsules in the first tensile process, which can be confirmed from the green traces following the tensile directions. The light green color in the images is the rejuvenator leaking traces out of microcapsules. After going through the healing cycles two times, as shown in Figure 11c, the rejuvenator traces have been widening because of the rejuvenator diffusion in bitumen. Mass transfer by molecular diffusion is one of the basic mechanisms in many branches of science. Molecular diffusion is a transport property that monitors the rate of mass transfer of species in a medium. The diffusion behaviors of bitumen have great relationships with temperature, viscosity of the diffusion medium, diffusant size and polarity [12,14]. 

**Figure 12:**
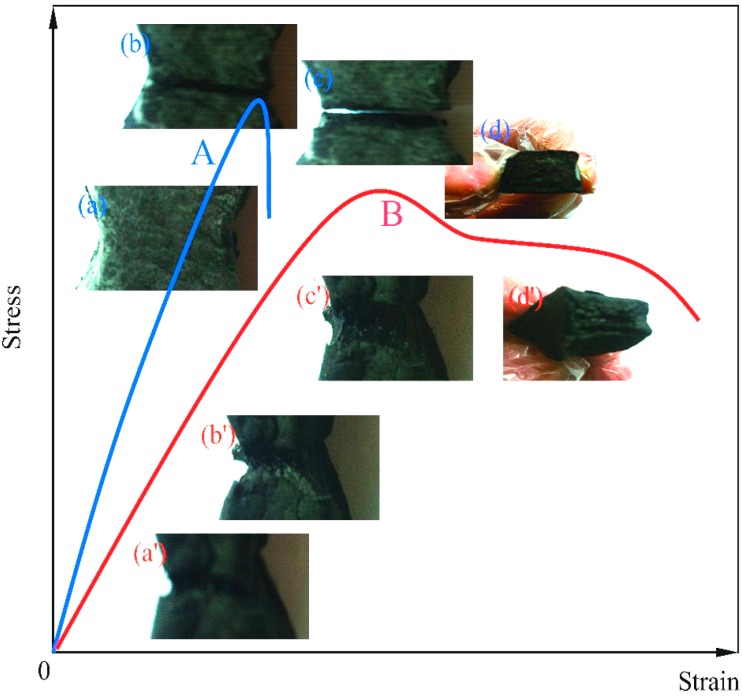
Tension fracture of microcapsules/bitumen samples: Images of bitumen samples broken process using a displacement speed of 100 mm·min^−1^ at 0 °C, (**a**–**d**) the pure bitumen (40/50), (**a**’–**d**’) the re-fracture of the same sample of bitumen (40/50) mixing with microcapsules (20 μm, 3.0 wt. %): **A** and **B** are the strain-stress curves.

The capillary and diffusion behaviors of rejuvenator also can be analyzed through the tensile fracture surfaces. Figure 12 shows the images of bitumen samples broken process using a displacement speed of 100 mm·min^−1^ at 0 °C. Curve A and curve B are the strain-stress curves of pure bitumen (40/50) and re-fracture sample of the microcapsules/bitumen (20 μm, 3.0 wt. %). Comparing the two lines, it can be known that the microcapsules/bitumen sample had enhanced the elastic-viscosity property. For pure aged bitumen (line A), it has less deformation under high stress. With the increasing of strain, a hard tensile break had appeared with straight sections of tensile fracture surfaces as shown in Figure 12a–d. After a capillary and diffusion process, the sample had a visco-elastic deformation form line B, which means that the sample had been softened. In Figure 12a’–d’, the tensile fracture surfaces are not regular with a tensile viscosity marks owing to long molecular deformation and creep. It indicates that the rejuvenator has penetrated into the bitumen molecules and improved the movement ability of bitumen molecules.

### 3.6. Mechanism Hypothesis of Self-Healing during Tension

Bitumen sample-mixing with microcapsules therefore induces multi-self-healing behavior as indicated by direct tensile tests. Morphological analysis confirms that the rejuvenator is released and penetrates into the aged bitumen. A mechanism *hypothesis* based on microstructure was given, which would help us to understand the self-healing principle more clearly, especially the microstructure of the broken bitumen surface.

**Figure 13:**
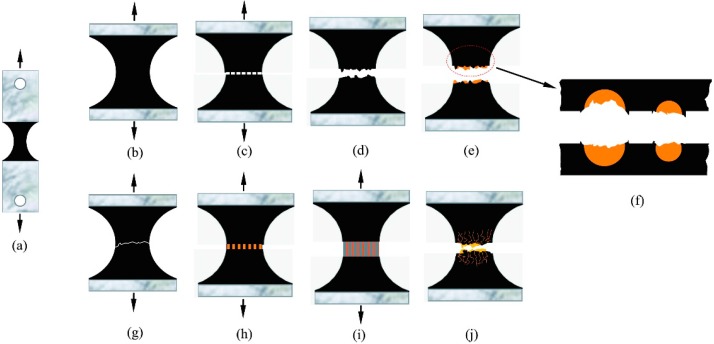
Illustration of self-healing of bitumen during tension test; (**a**) tension test sample; (**b**–**e**) the first-cycle of tension fracture; (**f**) the broken microcapsules on the interface of crack; (**g**) the healing process of bitumen sample; and (**h**–**j**) the second-cycle of tension fracture, rejuvenator penetration into aged bitumen.

A schematic is used to exhibit the micro-structural details of the self-healing bitumen during tensile tests. Figure 13a,b shows the original states of the tensile test sample. Arrows indicate the tensile strength directions. With increase in tensile strength, the sample experiences the first tensile-failure cycle as shown in Figure 13c–e. Given that the aged bitumen softening point is high, tensile failure produces a direct fracture with low elongation at the break. This result has been proven in the morphological analysis. Microcapsules have torn on the surface of the fracture section, and this results in a loss of strength, structure, and tightness (Figure 13f). The encapsulated oily rejuvenator is released and adheres to the surface. Then, the two broken pieces of the sample are reinserted into the module, while ensuring that the two parts fit the crack very well. Each sample in the module is heated at a certain temperature for 24 h as shown in Figure 13g. The crack closes completely, which does not imply the total recovery of re-fracture strength. Qiu [14] pointed out that once the crack is closed, bituminous samples may still contain micro-cracks and air bubbles inside the sample that are difficult to detect. During the second tensile fracture cycle, the tensile process is significantly different from the first tensile fracture cycle. Because of rejuvenator penetration into aged bitumen, oily agents can reconstitute binder chemical composition and consist of lubricating and extender oils that contain a high proportion of maltene constituents. The bitumen sample is softer as shown in Figure 13h,g. This increase in bitumen soft point also results from the increase in elongation at break. At the second tensile fracture (Figure 13j), the bitumen shows elastic behavior. The most important property in terms of elastomer mechanical behavior is that it has a three-dimensional network with physical chain entanglement or crosslinkage and interpenetration of molecules, which provides strength and elasticity. Poulikakos [13] reported on the failure phenomena of a viscoelastic thin film of bitumen under direct tensile tests, which confirms that flow in bitumen is dominated by viscous effects, although capillary forces do play a role at later stages of the experiment with negligible inertia and gravitational effects. This conclusion agrees with the phenomena observed in our tests, and that rejuvenator enhances the penetration and capillary nature of bitumen significantly.

Bituminous material is a visco-elastic polymeric material with a time-temperature-dependent behavior. A direct tensile strength analysis confirmed that higher temperature improves the healing speed. With the help of rejuvenator-containing microcapsules, the microcapsule/bitumen sample has a multi-self-healing ability. When its strength is lower than a limit point, the aged bitumen can no longer recover and its self-healing ability disappears. During the first cycle, aged bitumen recovers with the help of microcapsules that contain rejuvenator. Although the bitumen cannot return to its original state, the rejuvenator softens the aged bitumen. Microcapsules therefore only partly heal the bitumen. With increasing number of self-healing cycles, the extent of recovery decreases, which implies a loss of self-healing ability. The recovery time also increases with increasing number of self-healing cycles, which indicates that microcracks require more healing time with increasing aging degree of bitumen. Besides temperature, time also has a significant effect on healing ability. After an immediate reloading, the bitumen sample usually cannot return to the same point of the original loading state, but returns to a point with lower strength [2]. In this study, all samples had sufficient rest time before subsequent loading. Molecules in the interface therefore have sufficient time to regulate states or reactions, and the rejuvenator has sufficient time to move (capillarity and diffusion). An analysis of the recovery properties shows that crack healing can be considered to be a viscosity-driven process. However, crack closure does not imply total bitumen strength recovery. Improved healing may be achieved with a longer healing time.

## 4. Conclusions

The self-healing behaviors of bitumen using microcapsules containing rejuvenator had been investaged by a repetitive tension test. The tension strength values had been recoreded to evaluate the recovery efficiency of aged bitumen relative to original bitumen. Microstructure of bitumen/microcapsules samples were analyzed to help with understanding the healing mechanism. The following conclusions can be made:

1. The microcapsules can be broken by microcracks generating bitumen by a tensile strength; the oily rejuvenator can be easily penetrated and diffused into age bitumen.
2. A direct tensile test was applied to analyze the mechanical properties of bitumen/microcapsule materials. The results show that microcapsules of this sample can be broken by microcracks in different conditions with a good normal distribution. The bitumen has a multi-recovery ability. The size and content of microcapsules affects the tensile strength values of bitumen samples. The healing temperature and time greatly affect the healing process.
3. A reparative fracture-healing process of tensile tests confirms that the aged bitumen can have recovery properties with the help of rejuvenator.
4. The mechanism analysis indicates that the penetration and capillary behaviors of rejuvenator are the main reasons for self-healing of bitumen. The aged bitumen can be softened and have partial recovery properties with enough time under certain temperatures.

The above results will guide the optimization of the self-healing bitumen materials using microcapsules containing rejuvenator. In the future, the research will focus on the modeling of self-healing behaviors considering the parameters of size and content of microcapsules, healing time and healing temperature. In addition, a model will also be created to predicate the degree of self-healing and multi-self-healing. 

## Figures and Tables

**Table 1:** The microcapsule/bitumen samples with various microcapsules.

Samples	Core/Shell Ratio	Content (wt. %)	Mean Size (μm)
1	1:1	1.0	10 ± 0.2
2			20 ± 0.5
3			30 ± 0.4
4	1:1	3.0	10 ± 0.3
5			20 ± 0.5
6			30 ± 0.5
7	1:1	5.0	10 ± 0.6
8			20 ± 0.7
9			30 ± 0.5

**Table 2:** Properties of virgin and rejuvenated bitumen (penetration value, softing point value, viscosity value).

	Bitumen	Original Bitumen (80/100)	Aged Bitumen (40/50)	Tension Test Cycles			
Properties				1	2	3	4
Penetration (mm/10, 25 °C)		86	43	60	66	70	74
Softening point (°C)		46.7	53.5	52	49	48	48
Viscosity (135 °C) (mPa·s)		325	578	573	550	460	430
